# Preparation and Anticorrosion of Octadecyl Trichlorosilane SAMs for Copper Surface

**DOI:** 10.1155/2017/4975714

**Published:** 2017-12-14

**Authors:** Xue Shouqing, Liu Xiaohui

**Affiliations:** ^1^Institute of Fine Chemicals, Heze University, Heze, Shandong 274015, China; ^2^Department of Chemistry and Chemical Engineering, Heze University, Heze, Shandong 274015, China

## Abstract

The self-assembled monolayer (SAM) was prepared using octadecyl trichlorosilane (OTS) in distilled solution on the copper surface. The effect of inhibitor concentration on the rate of inhibition efficiency and corrosion rate in corrosion medium on copper by using polarization curves, electrochemical impedance spectroscopy (EIS), scanning electron microscope (SEM) was studied. The results showed that OTS SAMs exhibit the better corrosion resistance; the corrosion potential of copper OTS SAMs protection increased by about 1.02 V, while the corrosion current density decreased to 0.59 *μ*A/cm^2^. The corrosion rate is minimized and flattened and can reach 9.2% while the inhibition efficiency reached 95.4%, when the corrosion inhibitor has concentration of 40 ppm.

## 1. Introduction

With the rapid development of membrane technology, there are a lot of issues that need to be solved. Self-assembled monolayers (SAMs) [1, 2] are novel organic ultrathin films with close-pack, structured orientation, and highly superior order, which formed the basic structural units (atoms, molecules, nanoparticles, microns, or larger scale substance) in the gas, liquid, solid interface, and so forth, by chemical reaction or chemical adsorption which occurs between with the solid surfaces. This process is formed on different scale technical rules ordered structures under ambient conditions can be effectively designed with specific properties, highly ordered structure for a particular purpose composites [3, 4]. SAMs provide a simple, rapid, and convenient method of film under formation a mild condition, and thus the preparation technology has obtained a widespread concern.

Preparation method of SAMs is a spontaneous chemical process, with highly ordered structure and tropism, so it has higher requirements on metal surface. Currently, there are a large number of researches on stainless steel, aluminum, and other metals, because SAMs have stable and clean surface and cheap price. Raman and Gawalt [5] study the effect of water in the solvent on the corrosion inhibition performance of GPTMS SAMs on the surface of 430 stainless steel parts, and results indicated that the GPTMS SAMs obtained in absolute ethyl alcohol were more compact and had better corrosion resistance in solutions containing Cl^−^. Li et al. [6] conducted preparation on the surface of 2024 aluminum alloys by immersing method in the TDT solutions using mixtures of absolute ethanol with different volume ratios as the solvents. Results indicate that TDT shows a certain inhibition effect on the corrosion of aluminum alloys in Harrison solution, and the inhibition efficiency increases with the increasing of water content in the assembly solution.

However, copper and its alloys have been widely used in chemical industry, electronics, mechanical industry, construction, and other fields which is due to electrical properties, high strength, and excellent thermal properties [7, 8]. But the copper undergoes corrosion in the aqueous solution and atmosphere which limited its application.

Since 1980 Sagiv et al. produced monolayers on the glass surface with octadecyl trichlorosilane (OTS) SAMs, it has formed in a very active area of research in recent years, and some researchers attempted preparation of film on silane using different substrates, such as glass, ZnSe, and GeO_2_. But, so far, there are many research issues that need to be investigated in depth, and there are some issues such as zero lubrication and wear problem that still did not reach broad consensus. Therefore, this paper, using octadecyl trichlorosilane as raw material, successfully prepared functionalized SAMs in the copper surface and corrosion inhibition on copper was investigated by electrochemical impedance spectroscopy (EIS) SEM and PC technologies.

## 2. Experimental

### 2.1. Materials and Apparatus

Octadecyl trichlorosilane (OTS), AR, was purchased from Beijing Yun State Biotechnology Ltd.; and ethanol, AR, was detriment from Cymbalta Yi Chemical Co., Ltd.; hydrochloric acid, AR, was purchased from Aladdin Shanghai Biochemical Technology Co., Ltd.; copper sheet, 10 mm × 10 mm × 2 mm, was detriment from Dongguan Chenghui Hardware Factory.

### 2.2. Preparation of OTS SAMs

#### 2.2.1. Copper Sheet Pretreatment Process

The copper was cut into 20 mm × 20 mm × 1 mm square, and pretreatment was as follows.

The copper was repeatedly washed by secondary deionized water, then soaking water oscillating for 10 minutes, washed by acetone, ethanol, and secondary deionized water for 3–5 times, and dried, with irradiation for 40 min under UV. The treated copper was immersed in Piranha solution (H_2_SO_4_ : H_2_O_2_ = 70 : 30, V/V), for half an hour at 90°C, taken after repeated washing with deionized water. Then it was put into a mixed solution of ammonia and hydrogen peroxide for 10 min at 85°C, finally immersing mixed solution of hydrochloric acid and hydrogen peroxide at 85°C for 10 minutes, with repeated washing with deionized water, and dried with pure nitrogen, then using UV irradiation for 30 minutes.

#### 2.2.2. Preparation of OTS SAMs

The assembled silane solution was prepared with 50 mL of distilled benzene as a solvent, adding 2 drops of OTS. Then the copper was quickly immersed in the silane solution to stand for 3 min, after being removed, respectively, by benzene, acetone, and deionized water rinse. Finally, we performed drying by high purity nitrogen and then subjected it to an oven heat treatment at 120°C for 30 minutes.

### 2.3. Characterization and Electrochemical Measurements

The OTS SAMs corrosion inhibition was measured using the CHI660B electrochemical workstation. The electrolytic cell was a standard three-electrode system containing an auxiliary electrode which was a platinum electrode (area 2.25 cm^2^), reference electrode which was a saturated calomel electrode, and a smoothly polished copper sheet (20 mm × 20 mm × 1 mm) which was a working electrode; the concentration of the solutions is 0.02 mol/L and the scan rate is 2 mV/s. And the back of the working electrode by soldering copper, sealed with epoxy resin, with the exposed area of 10 mm × 10 mm. Corrosion morphologies of the copper sheet was observed by a TESCAN China Ltd. TESCAN analytical scanning electron microscope (SEM). All the measurements were performed at room temperature.

## 3. Results and Discussion

### 3.1. Effect of Inhibitor Concentration on the Rate of Inhibition Efficiency and Corrosion Rate

**Figure 1:**
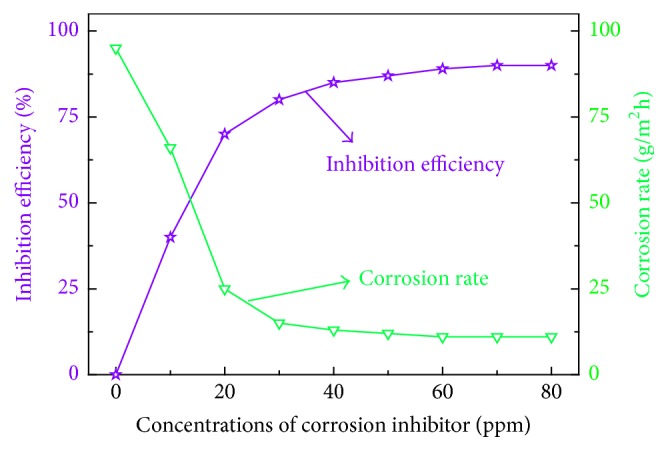
Effect of different inhibitor concentration on the rate of inhibition efficiency and corrosion rate.

Figure 1 showed the effect of different inhibitor concentration on the rate of inhibition efficiency and corrosion rate in 1 mol/L HCl solution. As can be seen from Figure 1, with the OTS concentration extended, the corrosion efficiency has been increased and the copper sheet corrosion rate has been reduced, when the concentrations of corrosion inhibitor can reach 40 ppm, the inhibition efficiency achieved was 95.4% while the corrosion rate curve is minimized and flattened. With further extension of the inhibitor concentration, there is no further enhancement of the inhibition efficiency and the corrosion rate is reduced (corrosion medium weightlessness with blank sheet of copper is 0.6789 g, corrosion rate is 29.7 g·m^−2^·h^−1^, while adding a 40 ppm corrosion inhibitor copper sheet weightlessness is 0.0217 g, and corrosion rate dropped to 0.87 g·m^−2^·h^−1^). Meanwhile, the copper test piece was visually coarse, with corrosion inhibitor added, the copper test piece surface brightness, and corrosion inhibitor class corrosion effect.

### 3.2. Polarization Curves of the OTS SAMs

**Figure 2:**
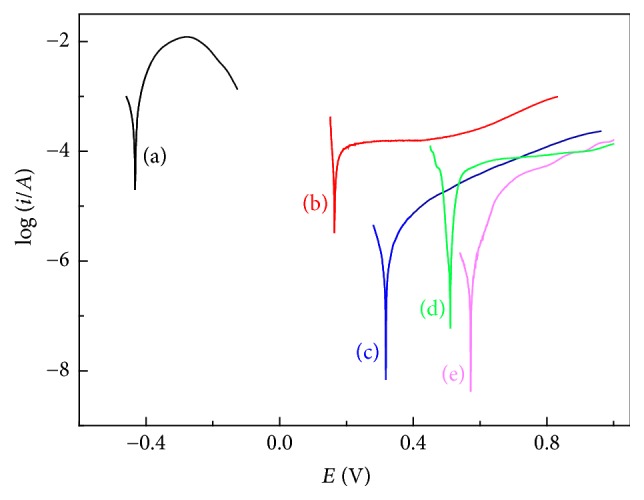
The polarization curves of copper and copper SAMs in 1 mol/L HCl solution. (a) Blank, (b) 10 ppm, (c) 20 ppm, (d) 30 ppm, and (e) 40 ppm.

Using of polarization curves to study the copper surface inhibition efficiency with OTS SAMs inhibition in 1 mol/L HCl.  Figure 2 showed the bare copper and copper/OTS SAMs film polarization curves in 1 mol/L HCl. Polarization curve fitting to give electrochemical parameters using *iA* = *i*_corr_[exp⁡(*ha*/*ba*) − exp⁡(−*hc*/*bc*)] was summarized in Table 1, with the potential voltage of −0.55~1.20 V and the scan rate of 2 mV/s. As can been seen from Figure 2 and Table 1, the corrosion potential of bare copper in 1 mol/L HCl is −0.44 V, while the corrosion current density is 3.18 × 10^3^ *μ*A/cm^2^. With adding of the 40 ppm corrosion inhibitor protected copper surface has higher corrosion potential that can go up to 1.02 V, while the corrosion current density decreased to 0.59 *μ*A/cm^2^. In addition, we can also espy that, with the use of OTS SAMs from 10 ppm to 40 ppm, the corrosion potential increases significantly. The OTS SAMs on anodic electrochemical processes have played a very good anti-inhibition role.

### 3.3. EIS of the OTS SAMs

**Figure 3:**
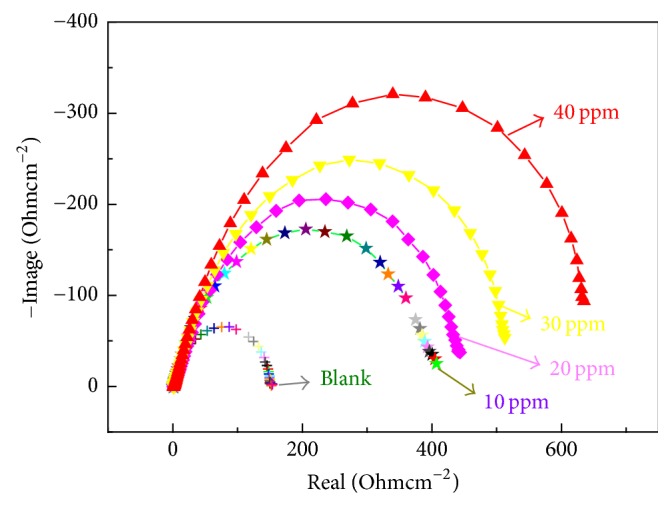
EIS of OTS/SAMs in 1 mol/L HCl.

Figure 3 shows electrochemical impedance spectroscopy of OTS/SAMs in 1 mol/L HCl solution. The impedance diagrams shows that, compared with bare copper, the high frequency region capacitive arc diameter significantly increased with the addition of OTS inhibitor, while the low frequency region Warburg impedance is gradually eliminated are lost. It because of that on the surface of copper emerged OTS SAMs, it can be effectively prevent Cl^−^ to contact with copper occur corrosion. With extending the concentration of inhibitor, high frequency region capacitive arc diameter sustained better performance, and when the inhibitor concentration is up to 40 ppm, OTS SAMs have few defects, which can effectively prevent copper avoiding occurrence of corrosion reaction.

### 3.4. SEM Characterization of the OTS SAMs

**Figure 4:**
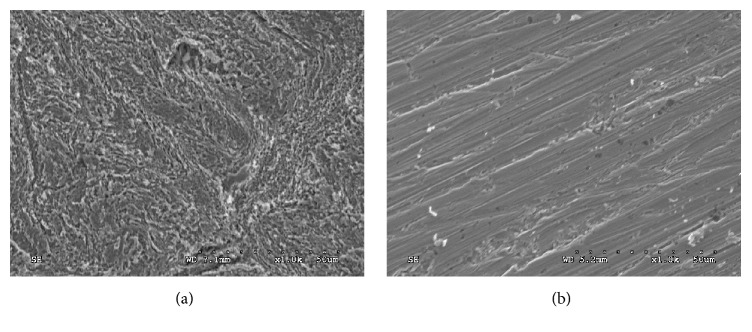
SEM characterization: (a) bare copper and (b) 40 ppm OTS/SAMs.

The surface micrographs of the specimens using JEM-2010 high resolution scanning electron microscopy were shown in Figure 4. As shown in Figure 4(a) without OTS SAMs that protected copper surface, the micrographs of copper surface underwent strong damage in HCl; and, adding the 40 ppm OTS SAMs, the corrosion reaction was efficiently inhibited (Figure 4(b)). These results insured the probability of using OTS SAMs as a good inhibitor to protect copper avoid.

## 4. Conclusion

We prepared the OTS SAMs in copper surface using electrochemical technology to study the OTS SAMs corrosion resistance. It showed that OTS SAMs have good corrosion resistance, and the corrosion potential of copper OTS SAMs protection increased by about 1.02 V, while the corrosion current density decreased to 0.59 A/*μ*A/cm^2^. When the corrosion inhibitor has concentration of 40 ppm, the inhibition efficiency reached 95.4%, while when the corrosion rate is minimized and flattened, it can reach 9.2%. These results prove that OTS SAM is a good inhibitor for protection of copper in acid medium.

## Figures and Tables

**Table 1:** The fitting parameters of polarization of copper and copper SAMs in 1 mol/L HCl solution.

Inhibitor concentration/ppm	*E*_corr_ (V)	*i*_corr_ (*μ*A/cm^2^)	*β*_*a*_ (V)	*β*_*c*_ (V)
Blank	−0.44	3.18 × 10^3^	0.060	0.039
10	0.18	134	0.112	0.056
20	0.33	76	0.101	0.003
30	0.45	2.3	0.085	0.054
40	0.57	0.59	0.025	0.056
